# The miR-16-1-3p passenger strand exhibits functional activity and suppresses malignant phenotypes in osteosarcoma

**DOI:** 10.3389/fgene.2026.1837827

**Published:** 2026-06-26

**Authors:** Wenyu Xue, Yuzhe Wang, Polina Pugacheva, Anna V. Smirnova, Roman Chuprov-Netochin, Margarita Pustovalova, Denis V. Kuzmin, Sergey Leonov

**Affiliations:** 1 Institute of Future Biophysics, Moscow Center for Advanced Studies, Moscow, Russia; 2 Phystech School of Biological and Medical Physics, Moscow Institute of Physics and Technology, Dolgoprudny, Russia; 3 Institute of Cell Biophysics of Russian Academy of Sciences, Pushchino, Russia

**Keywords:** CAM model, chemotherapy resistance, disease progression, miR-16-1-3p passenger strand, miRNA activity sensor, osteosarcoma

## Abstract

**Background:**

Chemotherapy resistance and disease progression remain major causes of mortality in osteosarcoma (OS). The miR-16 family is linked to tumors, but the specific function and treatment potential of the miR-16-1-3p passenger strand in OS remain unclear.

**Methods:**

RNA sequencing and clinical data from 82 OS patients in the TARGET-OS cohort were analyzed. Patients were stratified by progression status. Target genes of miR-16-1-3p were predicted using TargetScan and evaluated by gene set enrichment analysis (GSEA). Principal component analysis (PCA) and Kaplan–Meier survival analyses were performed based on cumulative Z-score–derived expression of the target gene set. Functional enrichment was assessed using Gene Ontology (GO) and Kyoto Encyclopedia of Genes and Genomes (KEGG) analyses. The impact of lentivirus-transduced miR-16-1-3p on U2OS cells was examined, focusing on proliferation, cell cycle, migration, colony formation, cisplatin sensitivity, and tumor growth in the CAM *in vivo* model. A fluorescent miRNA sensor was developed to assess intracellular target-binding functionality of the miR-16-1-3p passenger strand. Direct regulation of candidate target gene expression was validated using luciferase reporter assays and quantitative PCR. Expression of validated targets was further examined in a limited cohort of 15 chemotherapy-treated OS patients.

**Results:**

GSEA demonstrated significant enrichment of predicted miR-16-1-3p target genes among genes upregulated in patients with progressive disease. Kaplan–Meier analysis showed that elevated cumulative expression of predicted miR-16-1-3p target genes was associated with poorer overall survival in osteosarcoma patients. GO and KEGG analyses revealed enrichment of pathways related to translational regulation, mitochondrial function, and chemotherapy-associated signaling. A fluorescent miR-16-1-3p sensor system demonstrated sequence-dependent suppression of reporter activity following miR-16-1-3p overexpression. In functional assays performed in both U2OS and HOS cells, miR-16-1-3p overexpression significantly suppressed proliferation and migration and increased cisplatin sensitivity compared with control (scrambled miRNA) virus-transduced cells. Quantitative PCR and luciferase reporter assays further supported the interaction of miR-16-1-3p with SLC38A1 and ABCA13, two candidate genes potentially associated with chemoresistance.

**Conclusion:**

The miR-16-1-3p passenger strand exhibited functional activity associated with tumor-suppressive phenotypes and increased cisplatin sensitivity in osteosarcoma cells. Using integrative bioinformatics, fluorescent sensor assays, and functional experiments in U2OS and HOS cells points to miR-16-1-3p possibly regulating cancer characteristics and how osteosarcoma reacts to chemotherapy.

## 1 Introduction

Osteosarcoma (OS) is the most common malignant tumor of bone and joints, predominantly affecting children and adolescents, with a peak incidence during the second decade of life (Longhi et al., 2006; Ottaviani and Jaffe, 2009). Since the introduction of multi-target chemotherapy in the 1970s, the combination of surgical resection and adjuvant chemotherapy has markedly improved patient outcomes, increasing overall survival from less than 20% to approximately 70% (Ottaviani and Jaffe, 2009; Isakoff et al., 2015). Current standard treatment consists of neoadjuvant chemotherapy followed by surgical resection and postoperative chemotherapy, typically including cisplatin, doxorubicin, methotrexate, and ifosfamide (Zhang B. et al., 2020; Marchandet et al., 2021). However, despite these advances, long-term survival rates have plateaued over the past several decades, and further improvements remain limited (Bielack et al., 2008; Smith et al., 2014). Patients with metastatic disease continue to have a poor prognosis, with five-year survival rates of approximately 30% or lower (Tsuchiya et al., 2002; Kager et al., 2003; Mialou et al., 2005). Poor response to neoadjuvant chemotherapy, indicated by less than 90% tumor necrosis, is linked to worse long-term survival compared to good responders (Bielack et al., 2002). These clinical challenges underscore the urgent need to elucidate molecular mechanisms underlying OS progression and chemoresistance.

MicroRNAs (miRNAs) are small non-coding RNAs of approximately 20–22 nucleotides that regulate gene expression at the post-transcriptional level (Fang et al., 2013). Canonical miRNA biogenesis involves transcription of primary miRNAs, nuclear processing by the Drosha–DGCR8 complex, cytoplasmic cleavage by Dicer, and incorporation of one strand of the resulting duplex into the RNA-induced silencing complex (RISC), where it guides target mRNA repression through complementary binding to 3′ untranslated regions (3′-UTRs) (Wu, 2013; O'Brien et al., 2018; Medley et al., 2021). Traditionally, miRNA functionality has been attributed almost exclusively to the guide strand, while the passenger strand was assumed to be degraded and biologically inert. However, this paradigm has been challenged by accumulating evidence showing that passenger strands can be retained and exert regulatory activity in a context-dependent manner. Nevertheless, research on the functional importance of passenger miRNAs in tumor suppression and therapy response is still scarce, making their role in cancer biology unclear.

The role of miRNAs in regulating gene expression makes finding their target genes a major focus in cancer studies. Experimental methods for identifying miRNA targets are complex and often yield incomplete results. Bioinformatics tools such as TargetScan, miRDB, and miRanda are effective options because they use seed sequence matching along with evolutionary conservation (Betel et al., 2010; Huang et al., 2010; Agarwal et al., 2015; Wong and Wang, 2015; Riolo et al., 2020; Diener et al., 2024). These computational methods are crucial for analyzing extensive RNA-sequencing data from patients, as validating all candidate interactions experimentally is not practical.

Aberrant miRNA expression has been widely implicated in OS tumorigenesis, metastasis, and chemotherapy resistance (Zhang et al., 2007; Maximov and Aqeilan, 2016; Peng and Croce, 2016; Chen et al., 2019). Among tumor-suppressive miRNAs, the miR-16 family has been extensively studied for its anti-proliferative and chemosensitizing properties in OS (Maximov et al., 2019; Gu et al., 2020; Xu et al., 2021; Yu et al., 2021; Ghafouri-Fard et al., 2022). Most previous studies have focused on the canonical guide strand miR-16-5p, whereas the biological significance of the passenger strand miR-16-1-3p remains largely unexplored. MiR-16-1-3p is generated from the MIR16-1 locus on chromosome 13 and has been reported to possess regulatory activity comparable to, or in some contexts exceeding, that of the guide strand (Maximov et al., 2019). Notably, independent studies in lung cancer models have demonstrated tumor-suppressive and chemosensitizing functions of miR-16-1-3p, supporting the concept that passenger strands can act as functional regulatory miRNAs rather than inert by-products of miRNA biogenesis (Malakhov et al., 2025). However, the array of validated miR-16-1-3p target genes contributing to chemoresistance and OS progression have not been fully explored yet.

The present study attempted to enrich a functional annotation of miR-16-1-3p activity in osteosarcoma. By merging RNA-sequencing data and clinical annotations from TARGET-OS (Rothzerg et al., 2021), we assess how miR-16-1-3p contributes to chemoresistance and disease progression in OS through bioinformatics and experimental *in vitro* and CAM models.

## 2 Materials and methods

### 2.1 Bioinformatics analysis

RNA sequencing data of osteosarcoma patients were obtained from the TARGET-OS database. The original cohort consisted of 88 primary tumor samples with corresponding gene expression profiles. Principal component analysis (PCA) was first performed to assess data quality and identify potential outliers (Supplementary Figure S1). Samples showing marked deviation from the main population were excluded, resulting in a final cohort of 82 patients with complete expression and clinical follow-up information. These patients were subsequently classified into progressive disease (PD, n = 37) and progression-free survival (PFS, n = 45) groups according to clinical outcomes and were used for all primary downstream bioinformatic analyses. Raw RNA-seq data were normalized using the transcripts per million (TPM) method and log_2_-transformed [log_2_ (TPM + 1)] prior to analysis.

Predicted target genes of miR-16-1-3p were identified using the TargetScan 7.2 database, with genes exhibiting context++ scores < −0.2 considered high-confidence targets (Jacobsen et al., 2013; Patel et al., 2020). Differential expression analysis between PD and PFS groups was performed using the limma package in R, and ranked gene lists based on log_2_ fold change were generated. Gene set enrichment analysis (GSEA) was conducted using the fgsea package to evaluate enrichment of miR-16-1-3p target genes, with 1,000 permutations applied. Gene Ontology (GO) and Kyoto Encyclopedia of Genes and Genomes (KEGG) pathway enrichment analyses were subsequently performed using clusterProfiler, with adjusted p-values < 0.05 considered statistically significant. For each patient, expression values of target genes were Z-score–normalized and summed to generate a cumulative gene set expression score, representing the overall activity of the miR-16-1-3p target gene signature.

Survival analyses were performed by stratifying patients into high- and low-score groups based on the median cumulative gene set score. Kaplan–Meier survival curves were generated, and differences between groups were evaluated using log-rank tests. Receiver operating characteristic (ROC) analysis was conducted using the pROC package to assess the discriminatory ability of the cumulative expression score in distinguishing PD from PFS patients. Among the original cohort, chemotherapy treatment information was available for 17 patients; after PCA-based filtering (Supplementary Figure S1), 15 patients were retained and categorized into chemotherapy-resistant and chemotherapy-responsive groups. Due to the limited sample size, this subgroup was not included in the primary bioinformatic analyses but was used solely for supportive validation of known chemotherapy resistance–associated genes, including ABCA13 and SLC38A1.

### 2.2 Plasmids

The lentiviral backbone vector PLKO.3G was obtained from Addgene (plasmid #14748). Lentivirus packaging plasmids pLP1, pLP2, and pVSVG were purchased from Invitrogen (Thermo Fisher Scientific, United States). For fluorescent reporter assays, the mammalian expression vector pKatushka2S-C (cat.# FP761) encoding the far-red fluorescent protein Katushka2S was obtained from Evrogen JSC (Russia).

For functional studies, lentiviral constructs were generated to overexpress miR-16-1-3p or a scrambled microRNA control (Scr miR). The Scr miR sequence was earlier published elsewhere (Biton et al., 2011). Complementary oligonucleotides encoding miR-16-1-3p or Scr miR were annealed and cloned into the Acc36I and EcoRI restriction sites of the PLKO.3G vector. Correct insertion of all constructs was confirmed by PCR and Sanger sequencing.

To assess reporter repression associated with miR-16-1-3p overexpression, a fluorescent miRNA sensor construct was generated by inserting six tandem repeats complementary to miR-16-1-3p into the 3′untranslated region of a destabilized Katushka2S reporter gene. The destabilized reporter (dsKatushka2S) contains a degradation sequence derived from mouse ornithine decarboxylase, enabling rapid protein turnover and increased sensitivity to miRNA-mediated repression. Reduced fluorescence of the reporter construct was observed following miR-16-1-3p overexpression, suggesting a potential regulatory effect on reporter repression. This system enables real-time, single-cell-level visualization of reporter repression under experimental overexpression conditions. All oligonucleotide sequences used in this study are listed in Supplementary Table S1.

### 2.3 Cell culture, lentivirus production and cell transduction

Human U2OS, OS, and HeLa, cervical cancer, cell lines were obtained from Russian Cell Culture Collection of Vertebrates, Institute of Cytology, RAS, Russia. Human Embryonic Kidney 293 (HEK293T-CRL-3216) cell line was received from ATCC, United States. Cells were cultured in DMEM complemented with 10% heat-inactivated fetal bovine serum (FBS), 2 mM Gln, penicillin (100 I.U./ml), and streptomycin (100 μg/mL).

Lentiviruses were produced by co-transfection of each lentiviral plasmid (PLKO.3G-miR-16-1*and PLKO.3G-Scr miR, 3.55 µg) with packaging plasmids pLP1 (2.31 µg), pLP2 (0.89 µg), and pVSVG (1.25 µg) using the Lipofectamine 2000 transfection reagent (Thermo Fisher Scientific, United States) in HEK293T cells grown in 100 mm tissue culture plate at 80%–90% confluence. The cell culture medium with lentiviruses was collected in 72 h after transfections and filtrated through a 0.45 µm PES filter to remove the HEK293T cells.

To generate stable miR-overexpressing cell lines, human osteosarcoma U2OS cells were transduced with lentiviruses in a 6 well plates at 20%–30% of confluence by adding 2 mL of medium containing lentiviruses. Subsequently, EGFP-positive U2OS cells were sorted by the BIO-RAD S3e cell sorter (BIO-RAD, United States). The sorted EGFP-positive U2OS cells were utilized in the cisplatin sensitivity, colony formation, wound healing, transwell migration and in ovo tests. Successful overexpression of miR-16-1-3p in lentivirally transduced cells was verified by qRT-PCR (Supplementary Figure S2A).

For *in vitro* functional analyses, including Ki67 immunofluorescence staining, cell cycle assessment, and luciferase reporter assays, cells were transiently transfected with synthetic hsa-miR-16-1-3p mimics or a scrambled mimic (negative control). Transfections were performed using Lipofectamine™ 3000 (Thermo Fisher Scientific) in accordance with the manufacturer’s instructions. Cells were collected 24 h after transfection for subsequent analyses. Transfection efficiency and miR-16-1-3p overexpression were confirmed by qRT-PCR analysis (Supplementary Figure S2B).

### 2.4 Cell cycle analysis

Cell cycle distribution was assessed in cells transfected with hsa-miR-16-1-3p mimic or the corresponding scrambled control. At 24 h post-transfection, cells were harvested, rinsed with phosphate-buffered saline, and fixed in 70% ice-cold ethanol at 4 °C overnight. Fixed cells were then treated with RNase A (100 μg/mL) and stained with propidium iodide (50 μg/mL) for 30 min at 37 °C in the absence of light. Cellular DNA content was measured by flow cytometry (BD Biosciences), and the proportions of cells in G0/G1, S, and G2/M phases were determined using FlowJo software.

### 2.5 Cisplatin sensitivity test

The cisplatin sensitivity of miRNA-overexpressing U2OS cells was determined using the sulforhodamine B (SRB) assay (Vichai and Kirtikara, 2006). Briefly, cells were seeded in 96-well plates at a density of 10,000 cells per well and treated with serial dilutions of cisplatin (0.1–80 μM) for 48 h. After treatment, cells were fixed with 10% trichloroacetic acid (TCA) at 4 °C for 1 h. Fixed cells were washed with distilled water and stained with 0.4% SRB for 30 min at room temperature. Excess dye was removed by washing with 1% acetic acid, and the bound dye was dissolved in 10 mM Tris base (pH 10.5). Absorbance was measured at 540 nm using a microplate reader. The IC50 values were calculated applying the GraphPad Prism 8 software by fitting a non-linear regression curve to the dose-response data.

### 2.6 Colony formation assay

200 miR-overexpressing U2OS cells were seeded into each 10 cm culture dish and cultured in complete medium at 37 °C with 5% CO_2_ for 10 days. Then, cells were fixed with pre-chilled methanol for 15 min and stained with Giemsa solution for 30 min. Plates were rinsed with PBS, air-dried, and colonies were imaged and counted manually.

### 2.7 Wound healing (collective migration) assay

2D collective cell migration was assessed by wound healing assay. Serum-starved miR-overexpressing U2OS cells were seeded into 6-well plates and grown to approximately 90% confluence. A straight scratch was made across the monolayer using a sterile 200 μL pipette tip, and detached cells were removed by washing with PBS. Cells were then incubated in serum-free medium. Images of the wound area were captured at 0 h and 24 h by the EVOS™ M5000 Imaging System, and the scratch area was quantified using ImageJ software (National Institute of Health, United States). The migration rate was calculated as:

$$\left(\text{Initial wound area }–\text{Wound area at }24\;h\right)/\text{Initial wound area}\times 100\%$$

### 2.8 Transwell migration assay

Serum-starved miR-overexpressing U2OS cells were seeded at a density of 8 × 10^4^ cells per well into the upper chambers of Transwell inserts (8 μm pore size, 24-well format, Corning) containing serum-free medium. The lower chambers were filled with complete medium supplemented with 10% fetal bovine serum as a chemoattractant. After incubation at 37 °C in a 5% CO_2_ atmosphere for 24 h, cells remaining on the upper surface of the membrane were gently removed using a cotton swabs. Migrated cells on the lower surface were fixed with pre-chilled methanol for 15 min and stained with 0.1% crystal violet for 30 min. Following PBS washing, three random non-overlaping fields per well were imaged under an inverted microscope using ×10 magnification, and the amount of migrated cells were counted.

### 2.9 Fluorescent reporter assay for miR-16-1-3p-associated repression

To validate of miRNA biogenesis and activity within individual living cells, HEK293T and U2OS cells were seeded into 6-well plates and transfected at 60%–70% confluency using Lipofectamine 2000 (Thermo Fisher Scientific) according to the manufacturer’s instructions. Cells were co-transfected with a lentivirus constructs expressing miR-16-1-3p along with EGFP (PLKO.3G-miR-16-1*and PLKO.3G-Scr miR), and the Katushka2S reporter plasmid containing the miR-sensor sequence in its 3′UTR (dsKatushka 2S-miR-16-1-3p-Sensor). Forty-eight hours post-transfection, cells were harvested, washed twice with PBS, and subjected to flow cytometry. EGFP fluorescence served as a marker for successful transfection. The Katushka2S fluorescence intensity was assessed specifically in the EGFP-positive population, reflecting reporter repression observed under miR-16-1-3p overexpression conditions. Data analysis was performed using FlowJo software.

### 2.10 Chick embryo chorioallantoic membrane (CAM) *in vivo* assay

Fertilized specific pathogen-free chicken eggs were obtained from a certified hatchery (Trade house Ptichnoe, Ltd., https://ptichnoe-td.ru) and incubated at 37 °C with 70% relative humidity. On embryonic day 3 (EMD3), a small volume of albumen was removed to lower the chorioallantoic membrane (CAM), and a window was created in the eggshell to expose the CAM. The window was sealed, and eggs were returned to the incubator without rotation. On EMD8, tumor cells were implanted onto the CAM to assess the tumorigenic effects of miR-16-1-3p. A polytetrafluoroethylene (PTFE) ring was placed on a well-vascularized area, and 3 × 10^6^ U2OS cells suspended in a 1:1 mixture of serum-free medium and Matrigel (Corning, 356234) were seeded within the ring. Stable U2OS cells expressing miR-16-1-3p or scrambled miRNA served as the experimental and control groups, respectively. Following implantation, eggs were resealed and incubation continued. On EMD16, CAM tumors were excised and imaged using a stereomicroscope. Tumor mass was measured using an analytical balance, and Tumor volume was calculated by measuring the maximal diameter (L) and perpendicular height (H) with a vernier caliper and applying the ellipsoid formula: Volume (mm^3^) = (3/4) × π × (L/2)^2^ × H.

According to international legislations, chicken embryos are not classified as live animals before day 17 of incubation (Sarogni et al., 2021; Ribatti and Annese, 2023); therefore, all experiments completed by EMD 16 were exempt from IACUC approval.

### 2.11 Immunofluorescence staining

U2OS cells were transfected with hsa-miR-16-1-3p mimic or a scrambled negative control mimic and subsequently seeded onto sterile glass coverslips. Cells were fixed with 4% paraformaldehyde for 15 min and permeabilized with 0.2% Triton X-100 for 10 min. After blocking with 5% bovine serum albumin for 1 h at room temperature, cells were incubated overnight at 4 °C with a Ki-67 monoclonal antibody (SolA15, eBioscience™). Following washing with PBS, Alexa Fluor® 488-conjugated goat anti-rat IgG secondary antibody (1:500) was applied for 1 h in the dark. Nuclei were counterstained with DAPI, and images were acquired using the EVOS™ M5000 imaging system.

### 2.12 Gene expression qPCR

Total RNA was isolated from U2OS cells using ExtractRNA reagent (Evrogen, BC032). RNA concentration and purity were assessed using a NanoDrop 2000 spectrophotometer. To eliminate genomic DNA contamination, samples were treated with DNase I (Thermo Fisher Scientific) at 37 °C for 30 min, followed by enzyme inactivation at 65 °C for 10 min in the presence of EDTA. cDNA synthesis was performed using the High-Capacity cDNA Reverse Transcription Kit (Thermo Fisher Scientific, 4368814) with 10× RT Random Primers. The reverse transcription protocol consisted of incubation at 25 °C for 30 min, cDNA synthesis at 37 °C for 120 min, and enzyme inactivation at 85 °C for 5 min. Quantitative PCR was carried out on a CFX96 Real-Time PCR System (Bio-Rad) using SYBR Green PCR Master Mix (Thermo Fisher Scientific). Each 20 µL reaction mixture contained 2 µL of cDNA, 10 µL of SYBR Green Master Mix, 0.5 µL of forward and reverse primers (10 µM each) (see Supplementary Table 1), and 7 µL of nuclease-free water. The amplification program included an initial denaturation at 95 °C for 10 min, followed by 40 cycles of denaturation at 95 °C for 15 s and annealing/extension at 60 °C for 1 min, and a melt curve analysis from 65 °C to 95 °C. For normalization of gene expression, IPO8 was used as the reference gene based on its stable expression in this cell line. Relative expression levels were calculated using the ΔΔCt method.

### 2.13 Luciferase reporter assay

To evaluate the direct interaction between hsa-miR-16-1-3p and its predicted binding site, a luciferase reporter assay was conducted using the pISO dual-luciferase reporter vector (Addgene #12178), which is optimized for assessing microRNA-dependent regulation through 3′ untranslated region (3′-UTR) interactions. The predicted miR-16-1-3p target sequence (Supplementary Table S1) was amplified by PCR and inserted downstream of the firefly luciferase coding region within the 3′-UTR of the pISO vector, using MluI and XbaI restriction sites. Both the PCR product and the pISO plasmid were digested with the corresponding enzymes and ligated using T4 DNA ligase. Positive clones were identified by colony PCR and further confirmed by Sanger sequencing to ensure sequence fidelity. The construct harboring the wild-type target sequence was selected for subsequent analysis. Cells were co-transfected with the luciferase reporter plasmid and either miR-16-1-3p mimic or the corresponding negative control. Twenty-four hours after transfection, cells were lysed and firefly luciferase activity was quantified using a CLARIOstar microplate reader. A significant reduction in luciferase signal in the presence of miR-16-1-3p compared with the control was considered indirect evidence suggestive of a potential interaction between miR-16-1-3p and the predicted target sequence within the 3′-UTR.

### 2.14 Statistical analysis

Statistical comparisons were performed using Student’s t-test and one-way ANOVA in GraphPad Prism 10 (GraphPad Software, San Diego, CA, United States). Data are presented as mean ± SD from three independent experiments. A p-value < 0.05 was considered statistically significant.

## 3 Results

### 3.1 Gene set enrichment analysis of miR-16-1-3p target genes in osteosarcoma progression

Based on transcriptomic data from the TARGET-OS cohort, patients were stratified into a progressive disease (PD) group and a progression-free survival (PFS) group according to disease progression during follow-up. To evaluate whether the regulatory activity of miR-16-1-3p is associated with the progression phenotype, a candidate target gene set of miR-16-1-3p was first obtained using TargetScan prediction. Gene set enrichment analysis (GSEA) was then performed by ranking genes across the whole transcriptome according to differential expression between PD and PFS groups. This approach evaluates the positional shift of a predefined gene set within the global ranking, rather than relying on the significance of individual genes, thereby providing a more robust assessment of coordinated expression changes at the gene set level.

**FIGURE 1:**
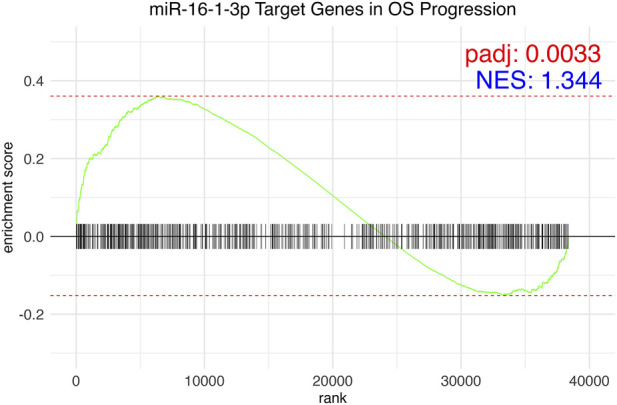
Gene set enrichment analysis (GSEA) of miR-16-1-3p target genes in the TARGET-OS cohort. NES and adjusted P value are indicated.

GSEA results demonstrated that the miR-16-1-3p target gene set was significantly enriched in the PD group, with the enrichment curve predominantly distributed toward the upregulated end of the ranked gene list. This finding indicates that, compared with the PFS group, samples from patients with disease progression exhibited higher overall expression levels of miR-16-1-3p target genes (Figure 1). Given that increased target gene expression is generally consistent with reduced miRNA-mediated repression, these results support an association between the expression pattern of miR-16-1-3p target genes and osteosarcoma progression.

### 3.2 Functional annotation of miR-16-1-3p target genes by GO and KEGG enrichment analysis

To further explore the potential biological functions associated with the miR-16-1-3p target gene set in osteosarcoma progression, Gene Ontology (GO) and Kyoto Encyclopedia of Genes and Genomes (KEGG) pathway enrichment analyses were performed using the progression-related miR-16-1-3p target genes.

**FIGURE 2:**
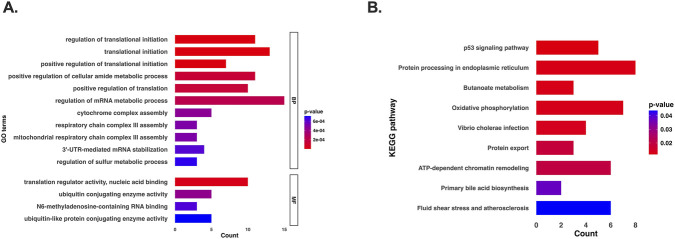
Gene Ontology (GO) and Kyoto Encyclopedia of Genes and Genomes (KEGG) enrichment analyses of progression-associated miR-16-1-3p target genes. **(A)** GO enrichment results showing representative biological process (BP) and molecular function (MF) terms. **(B)** KEGG pathway enrichment analysis of the same gene set. Bar length indicates gene count, and color represents adjusted P value.

GO biological process (BP) analysis revealed significant enrichment in multiple processes related to translational initiation and translational regulation, including regulation of translational initiation, translational initiation, positive regulation of translational initiation, and positive regulation of translation. In addition, terms associated with regulation of mRNA metabolism (regulation of mRNA metabolic process) and 3′UTR-mediated mRNA stabilization (3′-UTR-mediated mRNA stabilization) were also significantly enriched (Figure 2A).

At the molecular function (MF) level, the miR-16-1-3p target gene set was primarily enriched in functions related to translation regulatory activity (translation regulator activity, nucleic acid binding), ubiquitin-related enzymatic activities (ubiquitin-conjugating enzyme activity and ubiquitin-like protein conjugating enzyme activity), as well as RNA-binding functions, including N6-methyladenosine-containing RNA binding (Figure 2A). These results indicate a notable aggregation of miR-16-1-3p target genes at the level of post-transcriptional regulation and protein homeostasis.

KEGG pathway analysis further demonstrated significant enrichment of the miR-16-1-3p target gene set in pathways associated with cellular stress responses and energy metabolism, including the p53 signaling pathway, oxidative phosphorylation, protein processing in the endoplasmic reticulum, and ATP-dependent chromatin remodeling (Figure 2B). In addition, pathways related to metabolic adaptation, such as butanoate metabolism and primary bile acid biosynthesis, also showed enrichment trends.

Collectively, GO and KEGG analyses indicate that the miR-16-1-3p target gene set in progression-related samples is primarily associated with functional modules involving translational regulation, protein homeostasis, mitochondrial energy metabolism, and cellular stress response, providing functional-level support for its association with the osteosarcoma progression phenotype.

### 3.3 Construction of a cumulative expression score for the miR-16-1-3p target gene set and its association with clinical outcomes

To quantitatively evaluate the overall expression status of the miR-16-1-3p target gene set at the individual-sample level, a cumulative expression score was generated. Briefly, the expression values of selected putative miR-16-1-3p target genes were standardized by Z-score normalization across samples, and the normalized values were then summed for each patient. This composite score was used to represent the relative expression trend of the target-gene signature in each sample.

**FIGURE 3:**
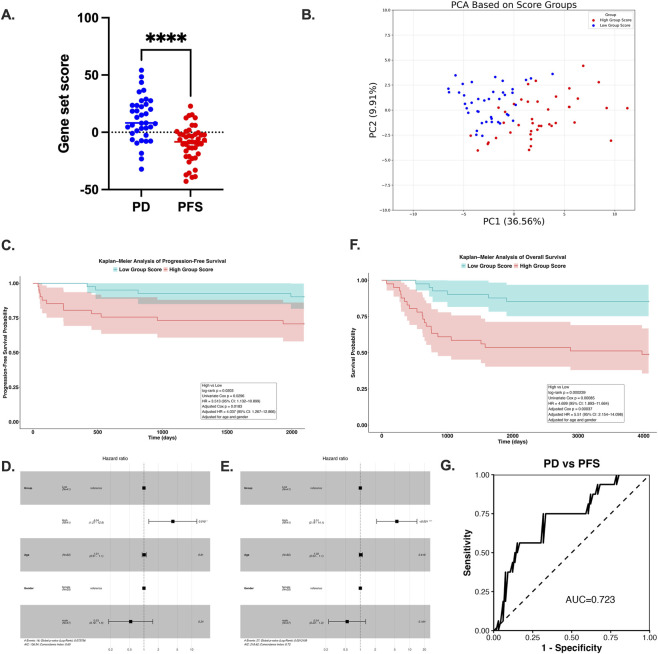
Clinical relevance of the cumulative miR-16-1-3p target-gene score in the TARGET-OS cohort. **(A)** Comparison of cumulative target-gene scores between patients with disease progression (PD) and patients without progression/progression-free status (PFS). Each dot represents one patient. Horizontal bars indicate mean ± SEM. ****p < 0.0001. **(B)** Principal component analysis (PCA) based on the expression matrix of the selected miR-16-1-3p target genes. Samples are colored according to predefined high-score and low-score groups generated using the median cumulative score. PCA was used as an unsupervised visualization approach and not for patient stratification. Percentages indicate the variance explained by each principal component. **(C)** Kaplan–Meier analysis of progression-free survival (PFS) comparing low-score and high-score groups stratified by the median cumulative score. Shaded areas indicate 95% confidence intervals. Statistical significance was determined by the log-rank test. **(D)** Forest plot showing hazard ratios (HRs) for PFS from univariable and multivariable Cox proportional hazards regression analyses, including adjustment for available clinical covariates. Error bars indicate 95% confidence intervals. **(E)** Forest plot showing hazard ratios (HRs) for overall survival (OS) from univariable and multivariable Cox proportional hazards regression analyses, including adjustment for available clinical covariates. Error bars indicate 95% confidence intervals. **(F)** Kaplan–Meier analysis of overall survival (OS) comparing low-score and high-score groups stratified by the median cumulative score. Shaded areas indicate 95% confidence intervals. Statistical significance was determined by the log-rank test. **(G)** Receiver operating characteristic (ROC) curve evaluating the performance of the cumulative score for distinguishing patients with disease progression from those without progression. The area under the curve (AUC) was 0.723, indicating moderate discriminatory ability within this cohort.

Comparative analysis across clinical groups showed that patients who experienced disease progression exhibited significantly higher cumulative scores than patients without progression (Figure 3A), suggesting that a higher cumulative score was associated with progression status in the TARGET-OS cohort.

Patients were subsequently stratified into high-score and low-score groups according to the median cumulative score. Principal component analysis (PCA) was then performed using the expression matrix of the selected target genes as an unsupervised visualization approach. The PCA plot showed a partial distributional separation between the two groups defined by the cumulative score (Figure 3B), suggesting that the cumulative score could, to some extent, reflect the overall expression characteristics of the selected target-gene set.

Kaplan–Meier survival analysis demonstrated that patients in the high-score group had significantly poorer progression-free survival (PFS) and overall survival (OS) than those in the low-score group (Figures 3C–F). To further assess whether this association remained after adjustment for available clinical covariates, multivariable Cox proportional hazards regression including age and sex was additionally performed. For OS, the adjusted hazard ratio (HR) for the cumulative score was 5.51 (95% CI: 2.154–14.098, p = 0.00037). For PFS, the adjusted HR was 4.037 (95% CI: 1.267–12.866, p = 0.0183). These results support the potential prognostic relevance of the scoring system within this dataset.

These findings suggest that a higher cumulative score was associated with poorer clinical outcomes in osteosarcoma. Receiver operating characteristic (ROC) analysis yielded an area under the curve (AUC) of 0.723, indicating a moderate discriminatory performance of the cumulative score for distinguishing patients with and without disease progression in this cohort (Figure 3G).

### 3.4 Validation of the target-binding activity of miR-16-1-3p using a fluorescent sensor system

**FIGURE 4:**
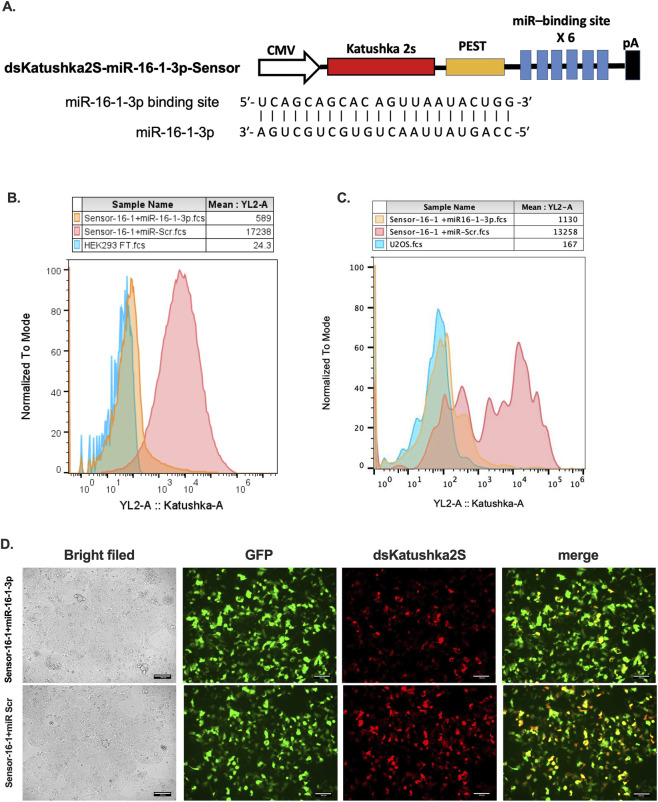
Detection of miR-16-1-3p activity using a fluorescent sensor in HEK293T and U2OS cells. **(A)** Schematic representation of the dsKatushka2S–miR-16-1-3p sensor construct containing tandem miR-16-1-3p binding sites in the 3′UTR of the reporter gene. **(B,C)** Flow cytometry analysis of Katushka2S fluorescence in HEK293T **(B)** and U2OS **(C)** cells co-transfected with the sensor plasmid and either miR-16-1-3p or scramble control. **(D)** Representative fluorescence microscopy images of HEK293T cells co-transfected with the sensor plasmid and either scramble control or miR-16-1-3p. Red fluorescence represents the dsKatushka2S sensor reporter, and green fluorescence indicates GFP-labeled miRNA expression. A reduction in red fluorescence intensity was observed in miR-16-1-3p–expressing cells compared with scramble controls. Scale bar = 100 μm.

To validate the target-binding activity of miR-16-1-3p, a fluorescent miRNA sensor plasmid (dsKatushka2S–miR-16-1-3p sensor) was constructed. This sensor contains six tandemly repeated sequences fully complementary to the seed region of miR-16-1-3p (nucleotides 2–8), which were inserted into the 3′ untranslated region (3′UTR) of the red fluorescent reporter gene dsKatushka2S (Figure 4A). Binding of miR-16-1-3p to these sequences is expected to induce translational repression or mRNA degradation, resulting in decreased red fluorescence intensity.

miR-16-1-3p and EGFP were co-expressed from the same plasmid, with EGFP serving as a marker for successful transfection. Red fluorescence intensity was specifically quantified within the GFP-positive cell population to assess miRNA-mediated repression, while GFP-negative cells were used as an internal background control.

In HEK293T cells, overexpression of miR-16-1-3p markedly reduced the mean fluorescence intensity (MFI) of the dsKatushka2S signal compared with the scramble miRNA control (Figure 4B). In contrast, the GFP-negative population exhibited only background-level fluorescence, indicating that the observed reduction was specific to miR-16-1-3p–mediated binding activity.

Consistent results were obtained in U2OS cells, where miR-16-1-3p overexpression also resulted in a clear decrease in dsKatushka2S fluorescence intensity relative to the control group (Figure 4C). These findings were consistent with reporter repression associated with possible regulatory activity of miR-16-1-3p in both non-tumor and osteosarcoma-derived cell lines.

In addition to flow cytometry analysis, fluorescence microscopy was performed in HEK293T cells to visually assess sensor repression at the single-cell level (Figure 4D). In scramble-transfected cells, strong red fluorescence from the dsKatushka2S reporter was observed in GFP-positive cells. In contrast, cells expressing miR-16-1-3p exhibited a noticeable reduction in red fluorescence intensity while maintaining GFP expression, indicating miRNA-mediated repression of the sensor construct. These imaging results were consistent with the flow cytometry findings.

EGFP fluorescence was used to gate miRNA-expressing cells. Katushka2S fluorescence intensity (YL2-A) was quantified to assess miRNA-mediated repression. Non-transfected cells were included as background controls. Representative histograms are shown.

### 3.5 Functional assessment of high miR-16-1-3p levels in osteosarcoma cell models

**FIGURE 5:**
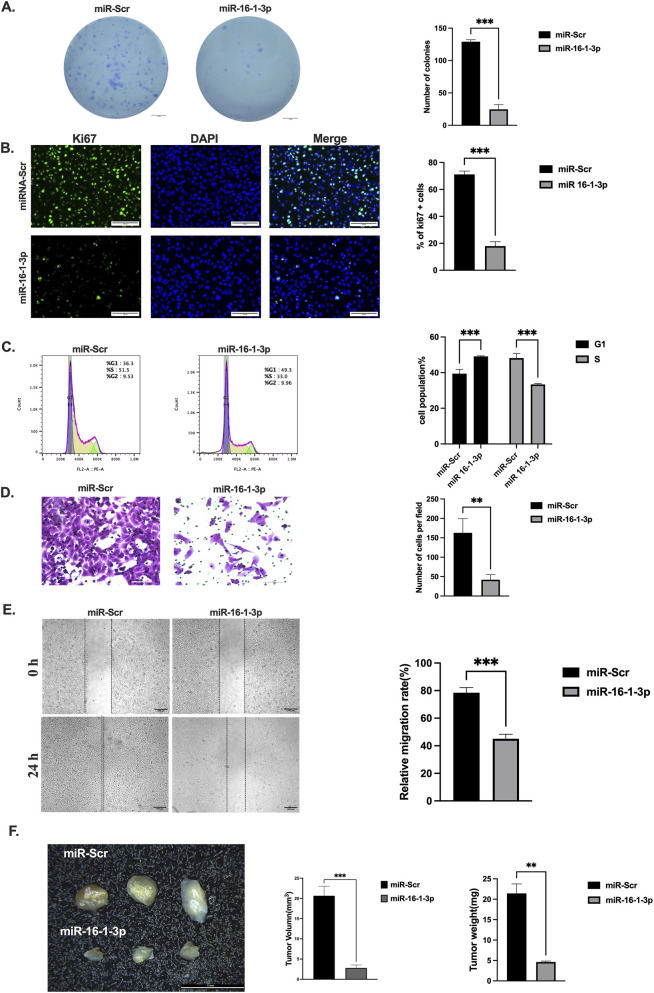
Functional effects of miR-16-1-3p overexpression. Progression-related phenotypes of miR-overexpressing U2OS cells **(A,D,E,F)** or U2OS cells that were transiently transfected **(B,C)** with synthetic miR-16-1-3p or scramble miRNA (miR-Scr) mimic at a final concentration of 10 nM. Transiently transfected cells were harvested 24 h after transfection for subsequent *in vitro* assays. Representative images are shown on the left, and quantitative analyses are shown on the right. **(A)** Colony formation assay assessing long-term self-renewal capacity. **(B)** Ki-67 immunofluorescence staining showing Ki-67 (green), DAPI (blue), and merged images. **(C)** Flow cytometric analysis of cell cycle distribution, with quantification of G1-and S-phase populations. **(D)** Transwell assay evaluating cell 3D confined migration, with representative images and quantification of migrated cells. **(E)** Wound-healing assay of 2D collective migration, showing representative images at 0 h and 24 h and quantification of relative migration rates. **(F)** Chick chorioallantoic membrane (CAM) model assessing tumor growth capacity of U2OS cells stably overexpressing miR-16-1-3p by lentiviral transduction. Representative tumor nodules and quantitative analysis of tumor volume and weight are shown on the right. Data are presented as mean ± SD. Statistical significance is indicated as: *p < 0.05; **p < 0.01; ***p < 0.001; ****p < 0.0001.

We increased miR-16-1-3p levels in U2OS cells to demonstrate its functional impact on osteosarcoma progression, employing increased scramble miRNA (SCR miR) as a benchmark. A series of cellular phenotypes closely related to tumor progression were systematically evaluated (Figure 5).

Colony formation assays were first performed to assess tumor cell self-renewal capacity. Compared with control cells, miR-16-1-3p-overexpressing cells formed significantly fewer colonies, indicating suppression of long-term self-renewal potential (Figure 5A).

To further evaluate proliferative activity at the population level, Ki-67 immunofluorescence staining was conducted. The proportion of Ki-67–positive cells was more than 3,5-fold reduced in miR-16-1-3p-elevated cells compared with controls (Figure 5B). Boosting levels of miR-16-1-3p resulted in significantly (p < 0.001) increased proportion of cells in the G1 phase and a corresponding significant (p < 0.001) decrease in the S-phase population relative to control cells, indicating a deceleration of cell cycle progression (Figure 5C).

Given that migration and invasion are critical biological features of osteosarcoma progression, their modulation by miR-16-1-3p was further examined. miR-16-1-3p-overexpressing cells displayed a markedly reduced (>2-fold, p < 0.001) 2D collective migration (wound closure) rate compared with controls under identical culture conditions (Figure 5D). Consistently, miR-16-1-3p-overexpressing cells possessed a significant reduction (>3-fold, p < 0.01) in 3D confined migration (the number of cells traversing the membrane) (Figure 5E).

To evaluate the effect of miR-16-1-3p on osteosarcoma growth *in vivo*, a chick chorioallantoic membrane (CAM) model was employed. Compared with control cells, miR-16-1-3p-overexpressing cells formed tumor nodules with significantly reduced volume (p < 0.001) and weight (p < 0.01) (Figure 5F).

In supplementary validation experiments using another osteosarcoma cell line, HOS, elevated miR-16-1-3p levels significantly impaired colony-forming capacity and reduced wound-healing and Transwell migration abilities (Supplementary Figure S3). These results were consistent with those observed in U2OS cells, further indicating that increased miR-16-1-3p levels were associated with less aggressive osteosarcoma phenotypes.

### 3.6 Evaluation of cisplatin sensitivity of miR-16-1-3p-overexpressing osteosarcoma cells

Our analyses indicated a close association between the miR-16-1-3p target gene set and osteosarcoma progression as well as unfavorable prognosis. Given the frequent clinical correlation between tumor progression and chemotherapy response, the effect of miR-16-1-3p on chemosensitivity was further evaluated. Mimics of either scrambled miR (miR-Scr) or miR-16-1-3p were transiently transfected in U2OS cells. Cisplatin sensitivity was assessed by determining its half-maximal inhibitory concentration (IC_50_).

**FIGURE 6:**
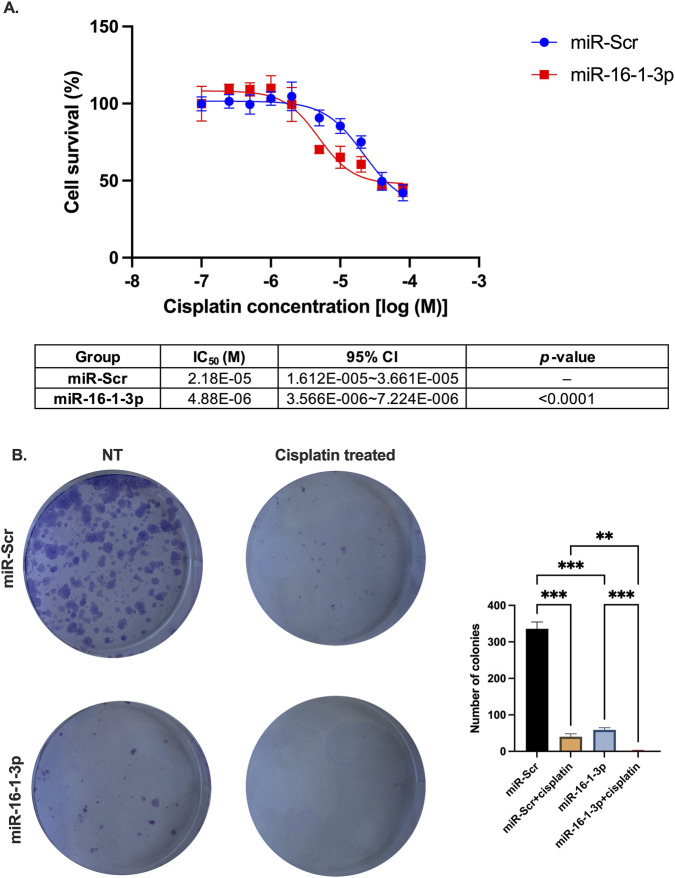
miR-16-1-3p overexpression increases cisplatin sensitivity in U2OS cells. **(A)** Cell survival (%) was assessed following cisplatin treatment at various concentrations (log [M]) using SRB assay. IC50 values are displayed in the table below the graph, indicating the differential sensitivity of each group. U2OS cells transfected with either hsa-miR-16-1-3p mimic (blue) showed significantly decreased IC50 compared with the negative control group transfected with scrambled miRNA (black), suggesting enhanced cisplatin sensitivity. **(B)** Representative colony formation images under control, miR-16-1-3p overexpression, cisplatin treatment, and combined treatment conditions.

Compared to control (miR-Scr) cells, miR-16-1-3p-transfected cells had a notably lower IC50 for cisplatin (Figure 6), demonstrating greater sensitivity to this drug.

In addition, while either miR-16-1-3p overexpression or cisplatin alone decreased colony formation compared with control cells, the combined treatment led to a more pronounced reduction in colony numbers. These findings further indicate that miR-16-1-3p overexpression increases the sensitivity of osteosarcoma cells to cisplatin.

Further experiments conducted in HOS cells demonstrated that increased miR-16-1-3p expression lowered the IC50 of cisplatin and enhanced the inhibitory effect of cisplatin on colony formation (Supplementary Figure S4). These findings paralleled those obtained in U2OS cells and further suggested that elevated miR-16-1-3p levels were associated with increased cisplatin sensitivity in osteosarcoma cells.

### 3.7 Exploratory analysis of candidate genes associated with miR-16-1-3p overexpression

To explore candidate genes potentially affected under miR-16-1-3p overexpression conditions and to evaluate their possible association with chemotherapy response, candidate genes were selected based on TargetScan prediction results together with previous reports linking them to drug response or unfavorable prognosis. Among these, the solute carrier family member SLC38A1 (Feng et al., 2023; Hushmandi et al., 2024) and the ATP-binding cassette (ABC) family of transmembrane transporter ABCA13 (Pasello et al., 2020; Tsyganov et al., 2022) have been reported to be associated with chemotherapy-related processes in several tumor types and were therefore included for further exploratory analysis.

**FIGURE 7:**
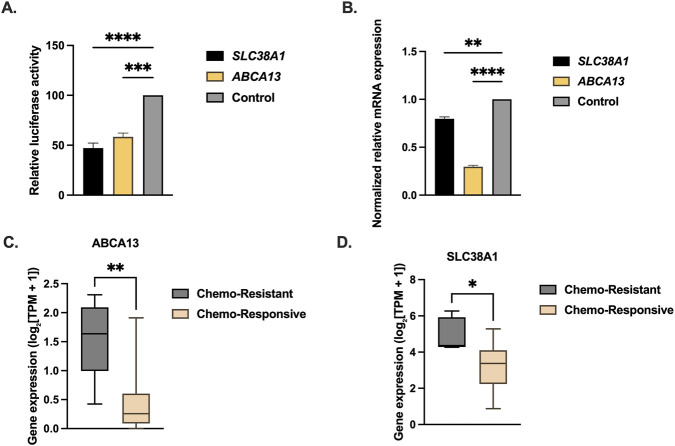
Exploratory analysis of candidate genes potentially associated with miR-16-1-3p overexpression and chemotherapy response. **(A)** Relative luciferase activity of reporters containing the 3′UTR of SLC38A1 or ABCA13 following co-transfection with miR-16-1-3p mimic or control miRNA in Hela cells. **(B)** Relative mRNA expression levels of SLC38A1 and ABCA13 after miR-16-1-3p overexpression. **(C,D)** Expression of ABCA13 **(C)** and SLC38A1 **(D)** in chemo-resistant versus chemo-responsive OS patients (log_2_ [TPM+1]). Data are presented as mean ± SD. *P < 0.05, **P < 0.01, ***P < 0.001, ****P < 0.0001.

Luciferase reporter constructs containing the 3′UTR sequences of either SLC38A1 or ABCA13 were generated and co-transfected with miR-16-1-3p or control miRNA into HeLa cells. This cell line was chosen because it is devoid of intrinsic miR-16-1-3p expression (Zubillaga-Guerrero et al., 2015). Luciferase assays showed that reporter activity was significantly reduced under miR-16-1-3p overexpression conditions (p < 0.001) (Figure 7A), suggesting a potential interaction between miR-16-1-3p and these 3′UTR regions. However, because mutant reporter constructs were not included in the present study, sequence-specific targeting could not be confirmed.

We next examined transcript levels of SLC38A1 and ABCA13 following miR-16-1-3p overexpression in U2OS cells. Compared with control cells, both genes showed reduced mRNA expression levels (Figure 7B), suggesting that they may represent candidate downstream genes associated with miR-16-1-3p overexpression. It should be noted that protein-level validation and rescue experiments were not performed in the current study. Therefore, the present findings should primarily be interpreted as functional observations obtained under overexpression conditions, while the precise mechanisms and endogenous biological relevance remain to be further investigated.

In addition, an exploratory analysis was performed in a limited number of chemotherapy-treated patients with available follow-up information. Compared with patients showing relatively favorable clinical outcomes, patients with unfavorable outcomes, including disease progression or death, exhibited relatively higher expression levels of SLC38A1 and ABCA13 (Figures 7C,D).

## 4 Discussion

Prior murine studies have shown that deletion of the miR-15a/miR-16-1 and/or miR-15b/miR-16-2 loci induces the development of multiple forms of leukemia and lymphoma (Klein et al., 2010; Lovat et al., 2015; Lovat et al., 2018). This observation suggests that mature miRNA strands derived from the miR-16-1/miR-16-2 loci may play a more prominent role during post-initiation stages of osteosarcoma progression rather than during tumor initiation. Consistent with this notion, the present study, based on a clinical cohort, suggests that the regulatory activity inferred from the overall expression pattern of the miR-16-1-3p target gene set is potentially associated with osteosarcoma progression phenotypes and unfavorable clinical outcomes, supporting the putative functional relevance of miR-16-1-3p during disease progression.

Previous studies have already indicated that miR-16-1-3p, traditionally regarded as a “passenger strand” generated during miRNA biogenesis, exhibits clear regulatory and functional potential in osteosarcoma. Although its expression level is generally lower than that of the guide strand miR-16-5p, miR-16-1-3p can participate in complex regulatory networks in a cell-context-dependent manner rather than serving merely as a degradation byproduct (Maximov et al., 2019). This observation is in line with accumulating evidence suggesting that miRNA regulatory effects are not strictly determined by expression abundance, and that a dissociation between miRNA “activity” and “expression level” may occur (Cheng and Li, 2008; Volinia et al., 2010; Liang et al., 2011; Schug et al., 2013; Zhang et al., 2014; Nielsen and Pedersen, 2021). Consequently, reliance on miRNA expression levels alone is often insufficient to explain their true biological functions, and inference of regulatory activity from target gene expression patterns represents a more functionally relevant analytical perspective. At the mechanistic level, miRNA activity is shaped by multiple regulatory layers, including RISC complex assembly and subcellular localization, post-translational modifications of Ago2 (such as phosphorylation) that affect RISC stability and function, and interference by RNA-binding proteins with specific miRNA–mRNA interactions (Liang et al., 2011; O'Brien et al., 2018; Mauro et al., 2023). In addition, increased abundance of target transcripts may competitively sequester miRNAs, thereby attenuating their regulatory effects on other targets, a phenomenon commonly referred to as the “sponge effect” (Mukherji et al., 2011). Highly expressed long non-coding RNAs (lncRNAs) and circular RNAs (circRNAs) may also function as competing endogenous RNAs (ceRNAs), leading to a decoupling of miRNA expression levels from actual regulatory activity (Hansen et al., 2013; Memczak et al., 2013). In the context of miR-16, several ceRNAs, including lncRNA TTN-AS1 and circRNA Circ-CUX1, have been reported to modulate miR-16 family activity (Zhang X. et al., 2020; Zhou et al., 2021), suggesting that miR-16-related strands in osteosarcoma may likewise be subject to complex post-transcriptional regulatory networks. Together, these findings provide a strong biological rationale for the strategy adopted in this study, namely, characterizing miRNA activity through target gene expression patterns.

Because direct measurement of miRNA activity remains technically challenging, an increasing number of studies have employed indirect approaches based on target mRNA expression profiles to evaluate miRNA functional status. In the present study, candidate target genes were predicted using TargetScan, and, based on the classical miRNA mechanism of action, elevated expression of target genes was interpreted as an indirect indicator of reduced miRNA-mediated repression in clinical samples. TargetScan, as a widely used high-throughput prediction tool, integrates seed sequence complementarity, binding free energy, and evolutionary conservation to provide a relatively reliable starting point for target identification in the absence of large-scale experimental validation (Agarwal et al., 2015). Nevertheless, as target prediction remains influenced by factors such as mRNA translational status and local structural context, integration with *in vitro* experimental validation remains essential. Notably, this study adopts a target-gene-set–level analytical framework rather than focusing on individual genes, an approach that has been demonstrated to be effective for predicting clinical outcomes and therapeutic responses in cancer patients (Wallden et al., 2015; Ohnstad et al., 2017).

In contrast to previous studies that primarily investigated the function of miR-16–5p in osteosarcoma cell lines, the present study focused on the potential functional relevance of miR-16-1-3p through integrative analysis of clinical transcriptomic data and experimental observations. Based on analysis of the TARGET-OS clinical transcriptomic cohort, the present study identified an association between inferred miR-16-1-3p activity derived from predicted target gene expression patterns and osteosarcoma progression phenotypes and survival outcomes (Figures 1–3). In addition, a fluorescent miRNA sensor system demonstrated sequence-dependent suppression of reporter activity following miR-16-1-3p overexpression (Figure 4), providing functional evidence consistent with the possibility that miR-16-1-3p exhibits regulatory activity.

We increased miR-16-1-3p levels in U2OS cells to demonstrate its impact on osteosarcoma progression, employing SCR miR as a benchmark. Functionally, increased levels of intracellular hsa-miR-16-1-3p suppress multiple malignant phenotypes associated with osteosarcoma progression *in vitro*. Compared with control cells, miR-16-1-3p-overexpressing cells formed significantly fewer colonies, indicating suppression of long-term self-renewal potential (Figure 5A), probably as a result of a slower cell cycle, which is supported by an increase in G1 phase cells and a decrease in S-phase cells in comparison to controls (Figure 5C).

It is now well accepted that proliferative states are commonly assessed through immunohistochemical analysis of proliferation markers, including Ki67, with proliferation recognized for many years as an important prognostic clinical indicator (Tubiana et al., 1984). However, recent data indicates that precise quantification of Ki67 antibody staining provides valuable insights that extend beyond merely determining whether a cell is in a proliferative state. This technique effectively distinguishes between rapidly cycling cells, which experience brief periods of quiescence, and those slowly cycling cells that remain in quiescence for significantly longer durations before eventually re-initiating the cell cycle (Miller et al., 2018). As such, the significantly reduced Ki67 staining in miR-16-1-3p-elevating cells (Figure 5B) can be interpreted rather as these cells appear to enter a state of halted cycling, potentially transitioning into quiescence in response to increased intracellular levels of miR-16-1-3p. The reduction in Ki67 expression in these cells suggests a potential quiescence-mediated survival strategy, which aligns with recent studies highlighting the importance of quiescence in cancer cell adaptation and drug resistance (Recasens and Munoz, 2019).

Noteworthy, Ki67 is not simply a marker of cell proliferation status; it also serves as an indicator of heterochromatin reorganization which, in turn, likely contributes to the remodeling of gene expression (Sobecki et al., 2016; Mrouj et al., 2021). Incidentally, our KEGG pathway analysis revealed significant enrichment of the miR-16-1-3p target genes in pathways linked to cellular stress responses and ATP-dependent chromatin remodeling (Figure 2B).

If that is the case, elevated levels of miR-16-1-3p may reduce Ki67-related changes in heterochromatin restructuring and gene expression remodeling during DNA damage responses like those caused by cisplatin. Furthermore, such reduction in heterochromatin reorganization-gene expression may explain the survival disadvantage of miR-16-1-3p-elevating cells when exposed to cisplatin insult. Indeed, the increased sensitivity to cisplatin (Figure 6) noted in our study may indirectly confirm this scenario, providing functional evidence for its potential involvement in DNA damage response regulation. Behind, such a gene expression remodeling may also responsible for impaired 2D collective (Figure 5D) and 3D confined (Figure 5E) migrations observed in our study. Moreover, cells with elevated miR-16-1-3p expression showed reduced tumor formation in the CAM model, providing additional functional evidence that the anti-tumor effects observed *in vitro* may also be reflected in an in vivo-like setting (Figure 5F). Together, these *in vitro* and CAM-based findings are consistent with the possibility that increased miR-16-1-3p levels contribute to the attenuation of malignant phenotypes associated with osteosarcoma progression, including cell proliferation, migration, colony formation, cisplatin responsiveness, and tumor growth capacity.

Given the frequent clinical overlap between tumor progression and therapeutic failure in osteosarcoma, consideration of progression-related clinical findings alongside *in vitro* chemosensitivity data provides a clinically relevant framework for interpreting the biological role of miR-16-1-3p.

This study explored several candidate genes potentially associated with miR-16-1-3p overexpression and chemotherapy-related phenotypes. Among these, ABCA13 and SLC38A1 were predicted by TargetScan as candidate miR-16-1-3p-associated genes and were further evaluated using reporter assays and transcript-level analyses (Figure 7). Luciferase reporter assays showed reduced reporter activity under miR-16-1-3p overexpression conditions, while mRNA levels of both genes were also decreased following miR-16-1-3p overexpression in U2OS cells. However, because mutant reporter constructs, protein-level validation, and rescue experiments were not included in the present study, direct sequence-specific targeting and mechanistic involvement could not be definitively validated. ABCA13, a member of the ATP-binding cassette transporter family, is linked to poor cancer prognosis and drug resistance. High levels of ABCA13 are associated with greater treatment failure (Hlaváč et al., 2013). Based on these previous observations together with our exploratory findings, ABCA13 may represent a candidate gene potentially associated with the functional effects observed under miR-16-1-3p overexpression conditions. SLC38A1 functions as a neutral amino acid transporter and has been implicated in tumor metabolism and stress adaptation. This observation is consistent with the KEGG enrichment analysis in the present study, which identified enrichment trends in pathways related to cellular stress responses and energy metabolism, including butanoate metabolism and primary bile acid biosynthesis (Figure 2B). Although direct evidence linking SLC38A1 to chemoresistance in osteosarcoma remains limited, amino acid transport and metabolic adaptation are increasingly recognized as important factors influencing tumor treatment responses (Januchowski et al., 2013). In the present study, SLC38A1 expression was relatively higher in patients with unfavorable clinical outcomes (Figure 7D), suggesting a possible association with treatment response–related phenotypes.

In summary, the present study provides functional observations consistent with the possibility that miR-16-1-3p may exhibit passenger-strand activity in osteosarcoma under the experimental conditions used in this study. Integrative analysis of TARGET-OS clinical transcriptomic data together with fluorescent sensor assays, *in vitro* functional experiments, and CAM model observations identified associations between inferred miR-16-1-3p activity, malignant phenotypes, and unfavorable clinical outcomes.

## 5 Conclusion

This study identified an association between inferred miR-16-1-3p activity and osteosarcoma progression phenotypes and unfavorable clinical outcomes in the TARGET-OS cohort. Under the study experimental conditions, overproducing miR-16-1-3p increased sensitivity to cisplatin *in vitro* and decreased tumor growth in osteosarcoma models *in vivo*. In addition, fluorescent sensor assays provided possibility that miR-16-1-3p may exhibit functional passenger-strand activity. Together, these findings warrant further investigation of miR-16-1-3p and its candidate downstream pathways in osteosarcoma progression and chemotherapy-related phenotypes. Additional validation in larger clinical cohorts and preclinical models will be necessary to further galvanize the biological and clinical relevance of these observations.

## 6 Limitations

This study has several limitations. First, the functional experiments primarily relied on miR-16-1-3p overexpression approaches, whereas complementary loss-of-function studies were not comprehensively performed. In addition, the present study did not directly evaluate endogenous miR-16-1-3p abundance relative to miR-16-5p using approaches such as absolute quantification or Northern blot analysis. Therefore, the endogenous biological relevance of miR-16-1-3p in osteosarcoma remains to be further clarified.

Second, although fluorescent sensor assays and functional experiments provided observations consistent with regulatory activity of miR-16-1-3p under the experimental conditions used in this study, direct evidence of Argonaute/RISC loading was not evaluated. Therefore, the physiological functional status of miR-16-1-3p as a passenger strand requires further investigation.

Third, only a limited number of osteosarcoma models were investigated. Given the substantial molecular and phenotypic heterogeneity of osteosarcoma, future studies using a broader range of cellular and animal models will be important to strengthen the generalizability of the present findings.

Fourth, candidate downstream genes were mainly supported by luciferase reporter assays and transcript-level analyses. However, protein-level validation, rescue experiments, and mutational validation of miRNA–target interactions were not included in the current study. Therefore, direct sequence-specific targeting and the precise mechanistic contribution of these genes to the observed phenotypes remain to be clarified.

Fifth, the bioinformatic analyses were based on retrospective public datasets. Although multiple statistical approaches were applied, the inferred miR-16-1-3p activity should be interpreted cautiously and requires validation in independent cohorts with matched miRNA and mRNA profiling data.

Finally, the CAM model provided supportive in vivo-like observations but does not fully recapitulate the biological complexity of mammalian tumor microenvironments. Further validation in conventional mammalian models will be important to better evaluate the biological and clinical relevance of these findings.

Overall, the present study primarily focused on clinical association analyses and functional observations under experimental overexpression conditions. Therefore, the proposed regulatory relationships should be regarded as predictive and exploratory, and their mechanistic validation will require more comprehensive and carefully designed future studies.

## Data Availability

The datasets analyzed in this study are publicly available from the TARGET-OS program through the Genomic Data Commons (GDC) Data Portal: portal.gdc.cancer.gov/projects/TARGET-OS.
