# Supplementary material for: The miR-16-1-3p passenger strand exhibits functional activity and suppresses malignant phenotypes in osteosarcoma
Source: Front Genet. 2026 Jun 26;17:1837827. doi: 10.3389/fgene.2026.1837827 (PMC13350050; doi:10.3389/fgene.2026.1837827)
Supplement: Supplementary file 1 [file Supplementaryfile1.pdf]

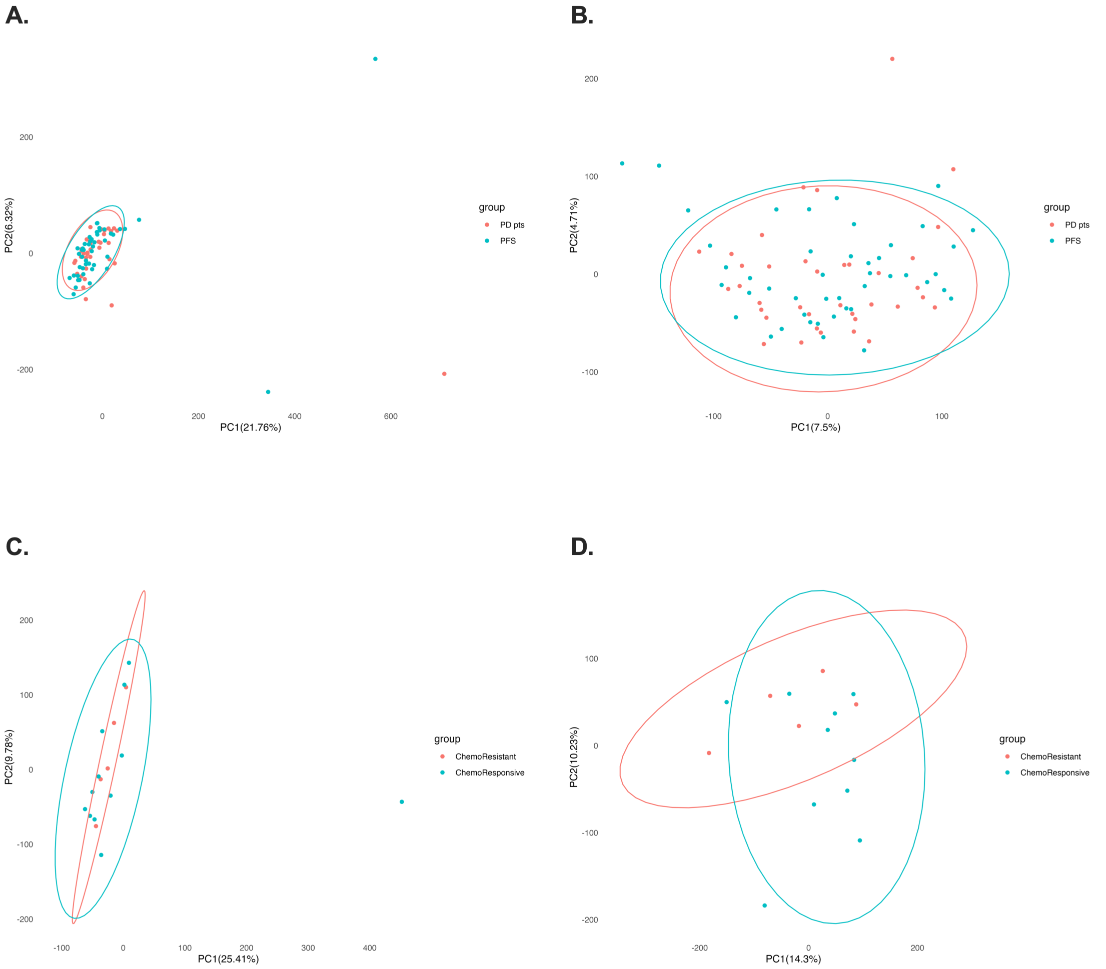

Supplementary Figure S1. Principal Component Analysis (PCA) for outlier detection in osteosarcoma datasets. (A) PCA of 88 osteosarcoma patients based on global gene expression profiles. (B) PCA of 82 patients after outlier removal. (C) PCA of 17 post-chemotherapy patients with available drug response data. (D) PCA of 15 patients after outlier removal. Ellipses represent 95% confidence intervals of sample clustering within each group.

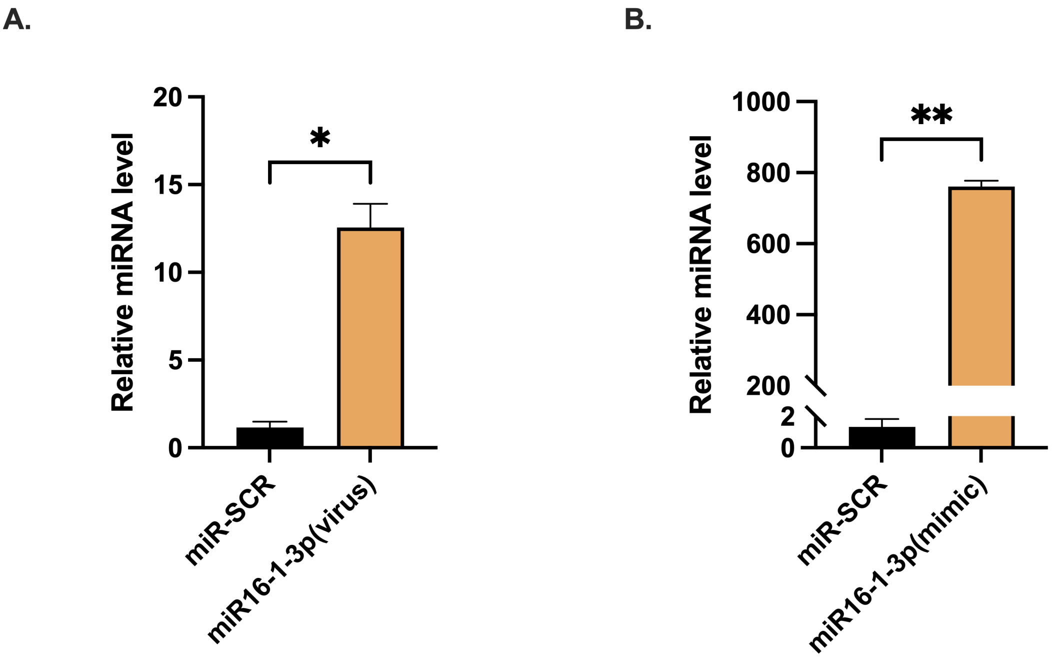

Supplementary Figure S2. Validation of miR-16-1-3p overexpression efficiency. (A) Relative miR-16-1-3p expression levels in U2OS cells stably transduced with lentiviral miR-16-1-3p or scrambled control (miR-SCR), as determined by qRT-PCR. (B) Relative miR-16-1-3p expression levels in U2OS cells transiently transfected with synthetic miR-16-1-3p mimics or scrambled negative control. Expression levels were normalized to U6 small nuclear RNA and calculated using the $2^{-\Delta\Delta Ct}$ method. Data are presented as mean ± SD from three independent experiments. $^{*}P < 0.05$, $^{**}P < 0.01$.

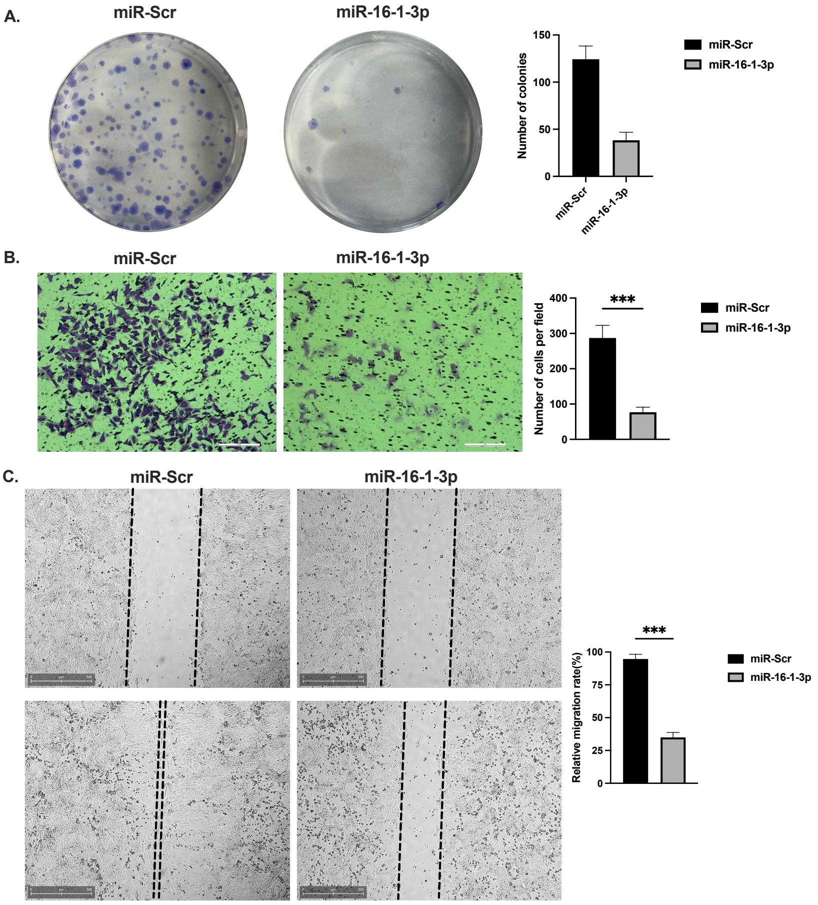

Supplementary Figure S3. Functional effects of miR-16-1-3p overexpression in HOS osteosarcoma cells.(A) Representative images and quantification of colony formation assays in HOS cells transfected with scrambled control (miR-Scr) or miR-16-1-3p mimic. Three independent biological replicates were performed for each group (n = 3). miR-16-1-3p overexpression significantly reduced colony-forming ability compared with the control group. (B) Representative images and quantification of Transwell migration assays in HOS cells transfected with miR-Scr or miR-16-1-3p mimic. Three independent biological replicates were performed for each group (n = 3). miR-16-1-3p overexpression significantly decreased the number of migrated cells. Scale bar = 50 μm.(C) Representative wound-healing images at the indicated time points and quantification of relative migration rate in HOS cells transfected with miR-Scr or miR-16-1-3p mimic. Three independent biological replicates were performed for each group (n = 3). miR-16-1-3p overexpression significantly impaired migratory capacity. Scale bar = 500 μm.

Data are presented as mean ± SD. P values were determined using two-sided Student's t-test. **P < 0.001.

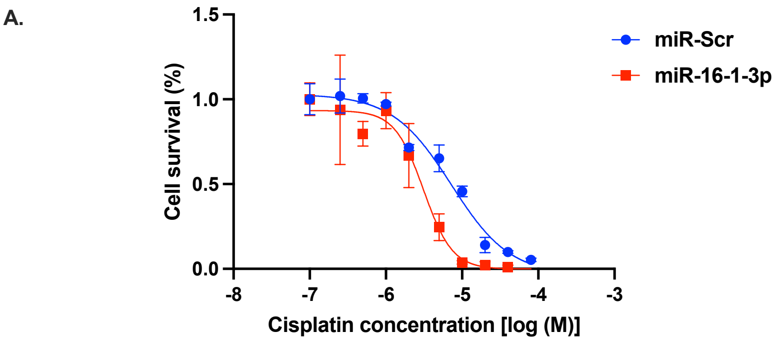

Group	IC_{50} (M)	95% CI	*p*-value
miR-Scr	7.23E-06	5.270E-006~1.076E-005	–
miR-16-1-3p	3.12E-06	2.299E-006~4.229E-006	0.0015

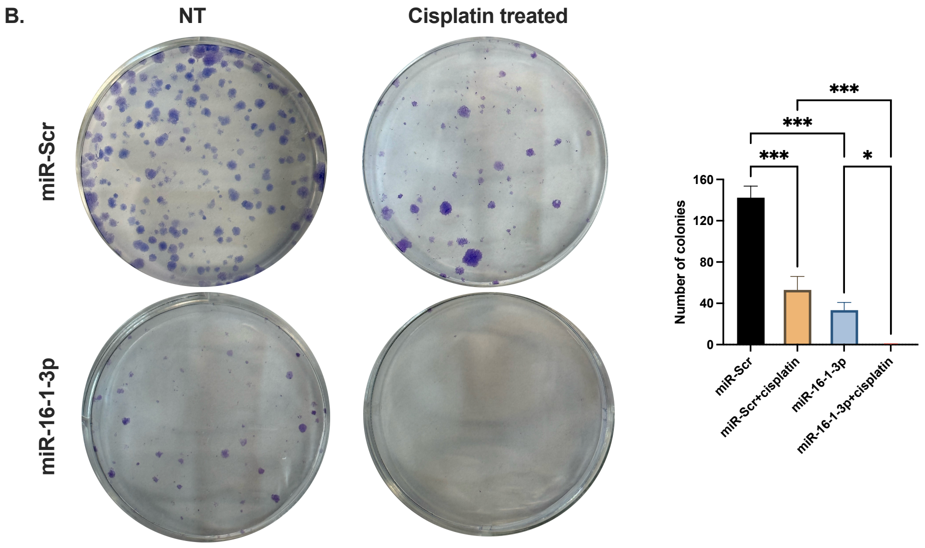

Supplementary Figure S4. miR-16-1-3p enhances cisplatin sensitivity in HOS osteosarcoma cells. (A) Dose–response curves of HOS cells transfected with scrambled control (miR-Scr) or miR-16-1-3p mimic following treatment with increasing concentrations of cisplatin. Three independent biological replicates were analyzed for each concentration (n = 3). miR-16-1-3p overexpression reduced the IC_{50} of cisplatin compared with the control group. IC_{50} values, 95% confidence intervals, and curve-comparison statistics are shown in the table. (B) Representative images and quantification of colony formation assays in HOS cells under the indicated treatment conditions (miR-Scr, miR-Scr + cisplatin, miR-16-1-3p, miR-16-1-3p + cisplatin). Three independent biological replicates were performed for each group (n = 3). Combined miR-16-1-3p overexpression and cisplatin treatment resulted in the strongest suppression of colony formation. Data are presented as mean ± SD. Statistical significance was analyzed using nonlinear regression curve comparison for IC_{50} analysis and one-way ANOVA with multiple-comparison testing for colony assays. $P < 0.05$, **$P < 0.001$.

Supplementary Table 1. List of oligonucleotides used in the article.

Oligonucleotide Name	Oligonucleotide Sequence
hsa-miR-16-1*-F	accggCCAGTATTAACTGTGCTGCTGActcgagTCAGCAGCACAGTTAATACTGGtttttg
hsa-miR-16-1*-R	aattcaaaaaCCAGTATTAACTGTGCTGCTGActcgagTCAGCAGCACAGTTAATACTGGc
shScrambled-F	aCCGGT*CCTAAGGTTAAGTCGCCCTCG*CTCGAG*CGAGGGCGACTTAACCTTAGG*TTTTTG
shScrambled-R	AATTCAAAAA*CCTAAGGTTAAGTCGCCCTCG*CTCGAG*CGAGGGCGACTTAACCTTAGG*Ac
PLKO-Dir	tgtggaaaggacgaaacacc
PLKO-Rev	tctttcccctgcactgtacc
SLC38A1-F	CTTTGGAGCCACCTCTCTACAG
SLC38A1-R	ACCAGGCTGAAAATGTCTCTTCC
ABCA13-F	GTACGTGTGCTCACCATCGTTG
ABCA13-R	AAGTGGTCCTGCGCCAAAGTGA
